# The Use of the Finite Elements Method (FEM) to Determine the Optimal Angle of Force Application in Relation to Grooves Notched into a Zirconia Coping with the Aim of Reducing Load on a Connection with Veneering Ceramic

**DOI:** 10.1155/2019/7485409

**Published:** 2019-07-01

**Authors:** Beata Śmielak, Leszek Klimek, Jacek Świniarski

**Affiliations:** ^1^Department of Dental Prosthetics, Medical University of Lodz, Ul. Pomorska 251, 92-213 Lodz, Poland; ^2^Department of Materials Research, Institute of Materials Science and Engineering, University of Technology, Ul. Stefanowskiego 1/15, 90-924 Lodz, Poland; ^3^Department of Strength of Materials, University of Technology, Ul. Stefanowskiego 1/15, 90-924 Lodz, Poland

## Abstract

**Objective of Study:**

To investigate, using the FEM, the influence of different notching angles on a zirconium dioxide coping with the aim of establishing the optimal connection conditions with veneering ceramic.

**Materials and Methods:**

To calculate the stresses in the connection between zirconia coping and veneering ceramic, a model comprising grooves cut perpendicular was adopted. Such a notch profile was used to design the shape and spacing of the grooves on an FEM model simulating a zirconium dioxide coping. For discretization purposes we used twenty-node solid BRICK elements featuring intermediate nodes with three degrees of freedom in each node. The model was divided into 117 745 finished elements and 439 131 nodes. The problem was solved with a GLU type contact. The same load** F** =** 1N** divided by the number of nodes on the external surface was applied to each node of the outer surface of the base. In subsequent computing variants the** F** load changed the orientation by angle **α** from** 0**° to** 45**° every** 15**°.

**Results:**

The highest level of material strain occurs at angle α = 0°  σ_red  max_ =309 MPa and the lowest at angle α = 45°  σ_red  max_ =220 MPa. The highest positive stress pressure occurs at angle α = 0°  p_max_=251 MPa, p_min_=-354 MPa and the lowest at angle α = 15°, p_max_=171 MPa, p_min_=-186 MPa. In the case of tangential stresses on the coping-veneering ceramic connection, the highest values were noted at angle α = 15°  τ_max_=44,4 MPa and the lowest at angle α = 45°  τ_max_=32,7 MPa.

**Conclusions:**

To reduce the load on the zirconia-veneering ceramic connection, the notches should be made at an angle of α = 45°.

## 1. Introduction

Despite their many advantages, zirconia-based restorations have poor connection parameters with veneering ceramic. More often than is the case with complex metal-porcelain structures veneering porcelain is at risk of chipping (15-62%), cracking (25-50%), delamination (> 10.7%), and major fractures (3-33%) [1–5].

The majority of authors are of the opinion that the quality of the connection between ceramic and zirconium dioxide depends on a suitably developed coping surface and the creation of microattachments between uneven areas of the base and the liquid ceramic [6, 7]. However, because of its considerable hardness, zirconium dioxide is more resistant to conventional machining than traditional ceramic and requires more aggressive action. The effects of acid etching are measurable on the nanoscale [8, 9]. In turn, abrasive blasting is a highly controversial approach [10, 11]. Fischer et al. report that abrasive blasting is not necessary at all [12]. Many studies reject the hypothesis that it significantly improves adhesion, regardless of the size of the aluminium oxide grains [13, 14]. Other studies point to the negative influence of sand-blasting on the structure of zirconium [15].

On one hand, other studies have demonstrated promising results for conditioning with the following lasers Nd:YAG, Er:YAG, CO2, XeCl, KrF, and ArF [16–19]. Various studies have compared the influence of different wavelengths, frequency, laser power, continuous wave mode, and pulse mode on roughness parameters and surface quality in SEM images. No one, on the other hand, has focused on the orientation of notches in relation to the load and its impact on connection strain. According to one study by Fischer et al., connection quality is dependent on the mechanical anchoring, the type and concentration of surface defects, and the level of stress on the ceramic layer [12].

The finite element method (FEM) allows us to establish the ideal conditions for surface development using a laser and assess connection quality after applying appropriate force. The FEM is a modern technique that is also currently widely used in scientific studies in the field of biomechanics. Taking into account the studies mentioned above, the question asked in the present paper is whether notches made with a diode laser should take the form of peripheral lines around the coping as well as lines running perpendicular to those already formed.

The aim of the present study was to find the optimal angle of force application in relation to the grooves notched into a zirconia coping, so as to reduce to a maximum the load on the connection between a coping and veneering ceramic.

## 2. Material and Methods

**Figure 1:**
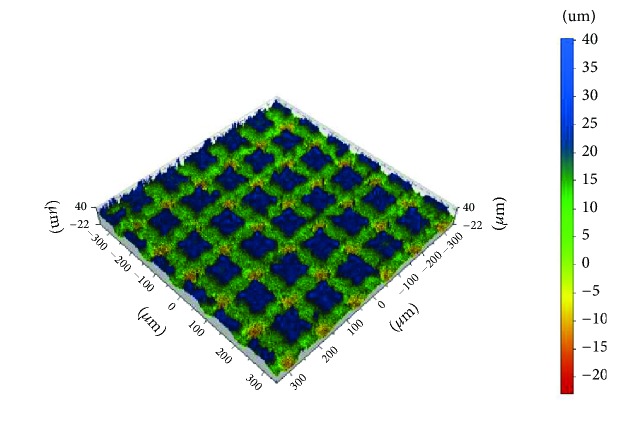
The shape and distribution of notches on a sample surface made from zirconia, notches in criss-cross pattern.

Ten rectangular 3Y-TZP Ceramill Zi sintered zirconia samples (Amann Girrbach AG, Koblach, Austria) with dimensions 10 x 10 x 5 mm underwent laser etching (Figure 1). One randomly chosen sample was selected for further testing. The choice of groove pattern was based on the results of previous studies in which the optimal values of the characteristic dimensions were determined [20]. To calculate the stresses in the connection between zirconia coping and veneering ceramic, we adopted a model comprising grooves cut perpendicular to one another with a width of 0.075 mm, a depth of 0.017 mm, and spacing of 0.115 mm. Such a notch profile was used to design the shape and spacing of the grooves on an FEM model simulating a zirconium dioxide coping.

The mechanical properties of the materials adopted for the FEM calculations are shown in Table 1.

The mechanical properties of materials used for FEM calculation purposes are presented in Table 1.

Table 1 is reproduced from [20] [under the Creative Commons Attribution License/public domain].

**Figure 2:**
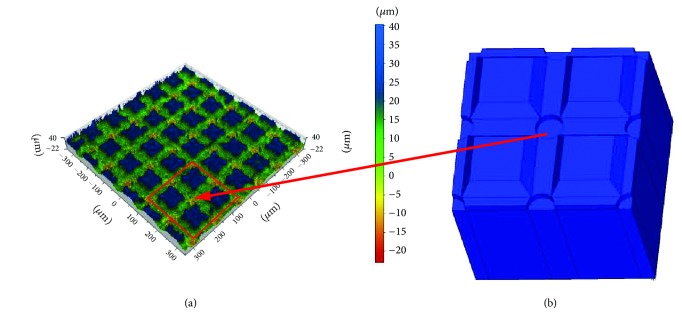
Shape and distribution of notches on the surface of the zirconium sample: (a) result of measurements; (b) FEM model.

**Figure 3:**
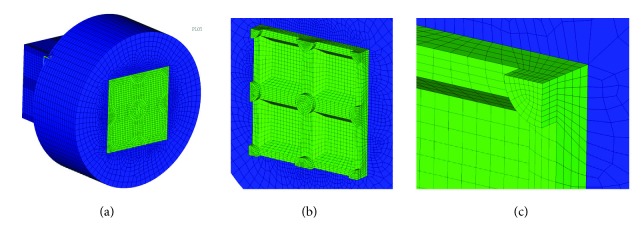
FEM mesh used for the following calculations: (a) isometric view of an FEM model, (b) FEM mesh of ceramic structure, and (c) enlarged corner of FEM mesh of ceramic structure.

Laser scanning microscopy showed that, at the point where the notches intersected, a crater had formed with an average depth of 0.01 mm as a result of the criss-crossing of laser beams during machining (this site can be said to have been machined twice, hence the recess). This crater was also taken into account in the adopted model Figures 2(b) and 3(b) as well as Figure 3(c). This crater is also taken into account in the adopted model. For discretization purposes we used twenty-node solid BRICK elements featuring intermediate nodes with three degrees of freedom in each node. The FEM model is presented in Figure 2. The zirconium dioxide coping is marked blue and the ceramic is marked green. The zirconium coping was supported in each direction in accordance with the symmetry conditions.

**Figure 4:**
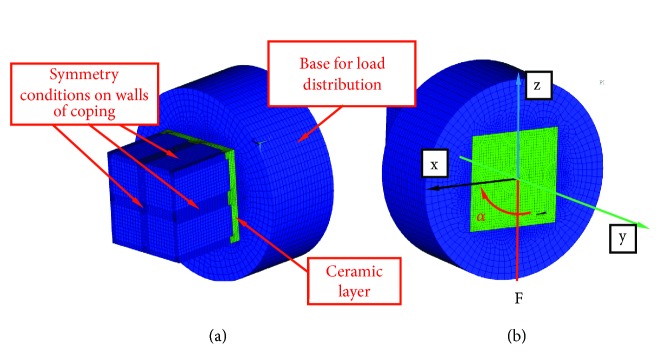
Boundary conditions adopted for calculations: (a) the way the model is supported; (b) the distribution of the load. Orientation of the load is changed from axis oz to axis ox in accordance with angle *α* shown.

The model was divided into 117 745 solid elements as well as 439 131 nodes. The FEM mesh was chosen in such a way that a twofold reduction in the size of the mesh would not cause a change in stress of greater than 2% between the zirconia and ceramic layers. This problem was solved by means of a BONDED type contact, i.e., with no possibility of separation, between the zirconia and the ceramic layer. The axial load was applied in the base shown in Figure 4(a) using RBE (RIGID BODY ELEMENT) elements in such a way that the same load F = 1N divided by the number of nodes on the external surface is applied to each node of the outer surface of the base. This type of load discretization corresponds to the even load of the hold along the entire circumference. In further calculation variants the F load changed the orientation by angle *α* from** 0**° to** 45**° every** 15**°. The location of the angle and load are shown in Figure 4(b).

## 3. Results

The results are presented in the form of colour maps of normal stresses, i.e., stresses perpendicular to the surface of the connection between the zirconia coping and the veneering ceramic, tangential stresses, i.e., the stresses lying on this surface, and reduced *σ*_red_ according to the Mises hypothesis. The results show the level of strain on the zirconia coping and the ceramic. They should be treated in a qualitative rather than a quantitative sense, as they show which material experiences more strain.

The colour code, ranging from navy blue to red in the computer print-out legend, illustrates the increase in stress values. Identical colouring in a given area of the mathematical model indicates approximately the same stress value at a given moment.

### 3.1. Results of Calculations

**Figure 5:**
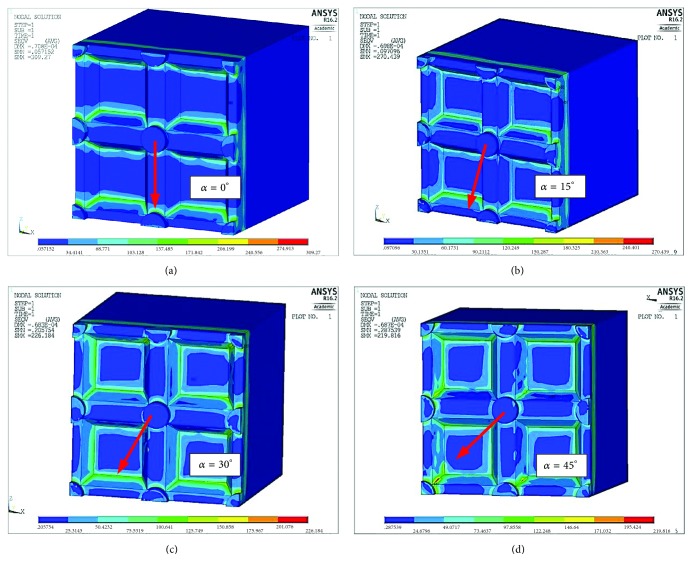
Map of stresses reduced according to the Huber (Mises) hypothesis depending on the angle of orientation of the applied load *α* in relation to the groove pattern. (a) *α* = 0°  *σ*_red max_=309 MPa, (b) *α* = 15°  *σ*_red max_=270 MPa, (c) *α* = 30°  *σ*_red max_=226 MPa, and (d) *α* = 45°  *σ*_red max_=220 MPa.

The results of the calculations for reduced stresses are compared with each other in the form of a map of reduced stresses according to the Huber (Mises) hypothesis and presented in Figure 5.

**Figure 6:**
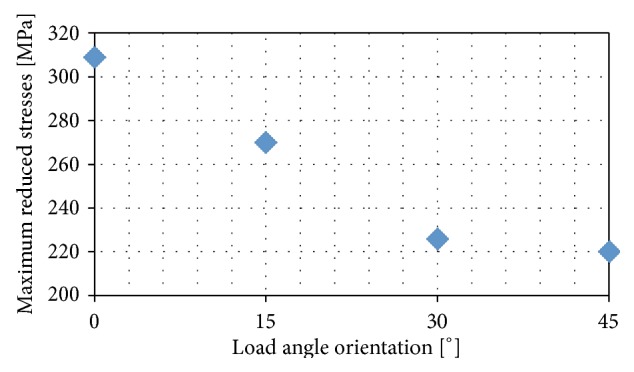
Maximum reduced stresses in ceramic as a function of the load orientation angle in relation to the groove pattern.

The results of these calculations are presented in Figure 6, so as to facilitate interpretation of the calculation results. It shows the maximum reduced strain as a function of the angle of load rotation.

The connection tightness may be disturbed by the ceramic breaking off from the base. This means that the adhesive bonds have become detached. The possibility of such a situation arising is illustrated by the distributions of pressure between the zirconia coping and the ceramic. The concept of normal pressure means that this value is always oriented perpendicularly to the contact surfaces of both materials, regardless of the orientation of the adjacent surfaces towards each other. In this case, our primary concern will not be the highest positive values, because here the ceramic presses onto the coping, but rather negative values (occurring on the opposite surface). In the latter scenario the ceramic breaks off from the coping and local layers of the connection are at risk of separating, which can lead to delamination.

**Figure 7:**
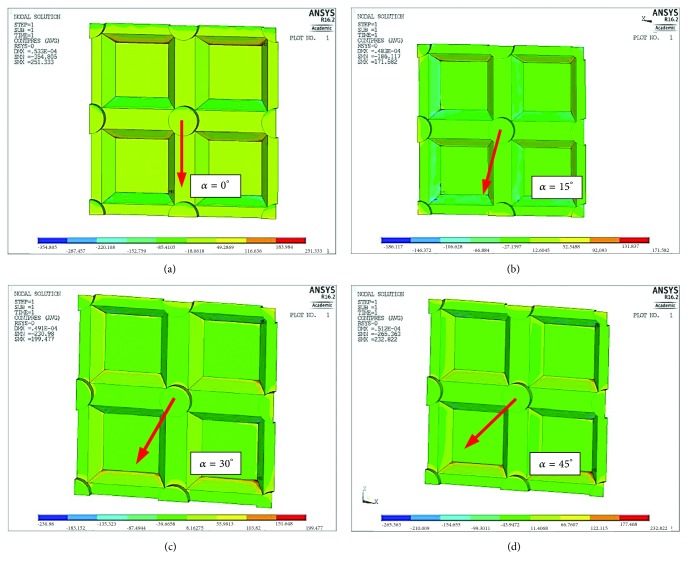
Map showing pressures between zirconia and ceramic (normal pressures) depending on the angle of load orientation *α*: (a) *α* = 0°  p_max_=251 MPa, p_min_=-354 MPa, (b) *α* = 15°  p_max_=171 MPa, p_min_=-186 MPa, (c) *α* = 30°  p_max_=199 MPa, p_min_=-230 MPa, and (d) *α* = 45°  p_max_=233 MPa, p_min_=-265 MPa.

Figure 7 shows the pressure distribution on the contact surface of the ceramic. A positive value denotes pressure on the connection while a negative value indicates that the connection has been broken. The results of the calculations are presented in Figure 7 in the form of a graph showing both pressure on the connection (a positive stress value) and detachment of the adhesive connection (a negative stress value).

**Figure 8:**
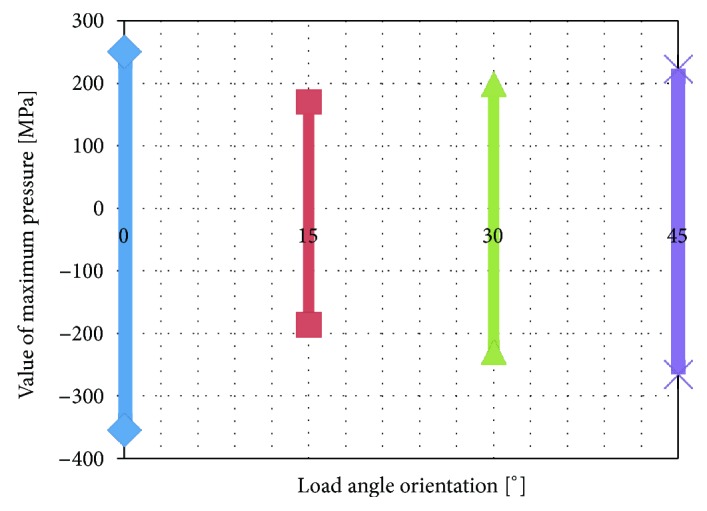
Distribution of pressures in contact with ceramic as a function of the angle orientation of the load in relation to a notched cross-section.

As Figure 8 shows, the greatest connection strain caused by surface pressures can be observed at load angle *α* = 0°. The lowest pressures were observed at angle *α* = 15°. The tensile stress break forces are lower at angle *α* = 45° than at angle *α* = 0° but greater than at angle *α* = 15°.

**Figure 9:**
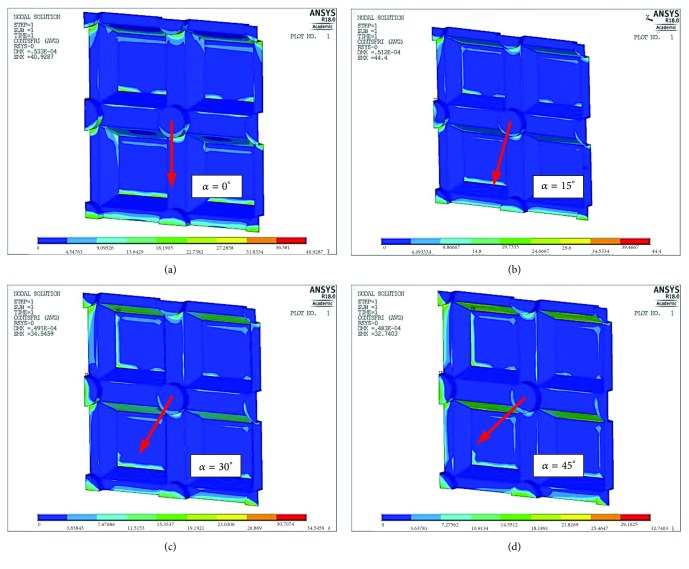
Map of tangential stresses between the metal coping and ceramic (shear stresses) depending on load orientation angle *α*: (a) *α* = 0°  *τ*_max_=41 MPa, (b) *α* = 15°  *τ*_max_=44,4 MPa, (c) *α* = 30°  *τ*_max_=34,5 MPa, and (d) *α* = 45°  *τ*_max_=32,7 MPa.

**Figure 10:**
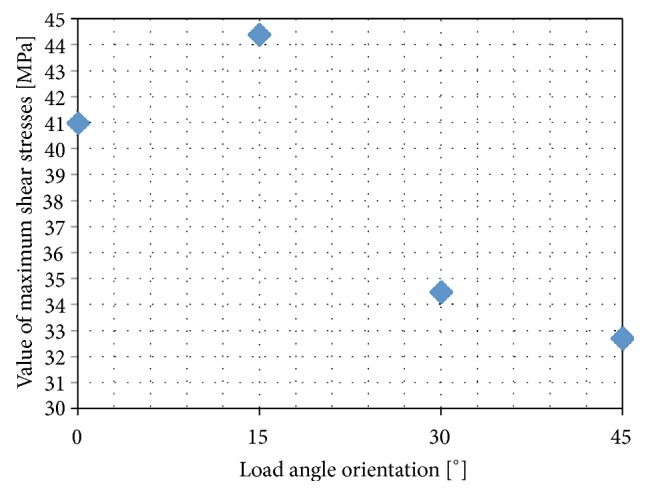
Distribution of maximum shear stresses in contact with ceramic, as a function of the angle orientation of the load in relation to the notched section.

In addition to the reduced stresses, corresponding in a straight line to the first signs of internal cracks in the ceramic, as well as the normal stresses responsible for the detachment of adhesive bonds, it is also worth paying attention to the shear stresses occurring in the connection surface between the ceramic and the zirconia coping. This type of stress is particularly dangerous because in addition to thermal deformations it can initiate delamination between the layers, which when combined with normal stresses can quickly result in the ceramic breaking off from the coping.  Figure 9 shows the distributions of tangential stresses on the surfaces of the connection between the zirconia coping and the veneering ceramic, whereas Figure 10 shows the distribution of these stresses as a function of the angle of load application in relation to the arrangement of the grooves.

As can be seen in this case, shear stresses were highest where minimum normal stresses were observed (*α* = 15°). From Figure 10 it can be seen that, just as with the reduced stresses, the least strained connections resulting from shearing forces were observed at angle *α* = 45°.

## 4. Discussion

The FEM allows us to study structures with complex shapes and composed of various materials and subjected to any* loads*, without the need for their physical construction or loading. The tests are carried out on computer models of the examined objects. The accuracy of the calculations depends on the number of elements that the model is divided into. To achieve credible results the model should be divided into a large number of elements, which at the same time increases the degrees of freedom and, as a consequence, significantly extends the computation time [21]. Besides this, the boundary conditions, especially in terms of loading, should be very carefully analysed.

In the presented FEM tests, it was assumed that there was an ideal connection between the different materials and that it remained undamaged despite the increase in loading. In clinical reality, no ideal connection exists. This is due to inaccuracies in laboratory-made connections [22].

The purpose of the calculations was to determine reduced stresses in ceramics, the pressure between a zirconia coping layer and the veneering ceramic, and shear stresses occurring at the boundary between the coping and the veneering layer.

The notches made on the coping surface and the placement of the veneering material in these places were aimed at reducing the load on the adhesive connections between the ceramic and the coping to facilitate mechanical load transfer (mechanical lock). This in turn was designed to ensure the combination of both materials was not purely adhesive in character but rather mechanical-adhesive.

To ensure the complete and correct transfer of the load via mechanical phenomena such as shearing of the coping or the veneering ceramic, the grooves on the zirconia coping should have walls set perpendicular to the contact surface of both materials, which would offer the best solution for the connection. Then, instead of shearing the adhesive layer the lateral forces would be transferred through the volume of material present in the created groove. However, the use of laser technology to make grooves makes it impossible to construct groove walls for the coping and veneering ceramic that would run perpendicular to the contact surface. There are also limitations regarding their size and depth.

If a clean mechanical connection cannot be achieved, the inclined groove walls are loaded with surface pressures resulting from the shape of the connection as well as with shearing forces of the ceramic-coping connection. It is well known that a flat connection only has the strength of an adhesive connection. A mixed adhesive-mechanical connection partially eliminates the risk of adhesive connection detachment by transferring loads via microattachments, i.e., “locks” formed by the notches in the coping surface. However, there is a tendency for stress to accumulate in the corners of grooves as a result of transferred mechanical loads. The accumulation or otherwise stress concentrations or material strain only affects small areas. In the case of an actual nonidealized surface, we can assume that these concentrations will be blurred by mechanical microlocks (irregularities on the processed surface).

It is important to stress that the calculation results presented here are qualitative and not quantitative in character. They show which elements are subject to most strain. For computation purposes a loading force of 1N was adopted. The application of a different force will of course change the stress values, but it will not alter the relationship between them. Not all aspects can be modelled according to the FEM calculation, because there is no table with critical stress values for the adhesion and for the shearing of the bond.

The calculations show that when it comes to reduced stresses, the most advantageous situation is one where the loading force is directed at an angle of 45° in relation to the groove system (Figure 5(d)) and this arrangement is the most effective in terms of the material load. However, most often damage to the coping-veneering ceramic connection occurs within the interface between these two materials and therefore an analysis of contact pressures and shear stresses is more important. As far as pressure is concerned, the most favourable conditions arise when the load is applied at an angle of 15° to the groove system (Figures 7(b) and 8). However, an analysis of the tangential stresses responsible for the delamination of the connection shows that these forces are highest at an angle of 15° and lowest at an angle of 45°. Summing up, it appears that the most favourable load system is one where the loading force is applied at an angle of close to 45° in relation to the groove system.

## 5. Conclusions

1. A change in the orientation of the notches in relation to the load reduces connection strain and improves connection stability.
2. It has been demonstrated that the notches should be made at an angle *α*= 45° so as to reduce stress concentrations and thus minimise the stress occurring in the corners of the connection.
3. It is recommended that cuts be made in two mutually perpendicular directions so as to make orientation independent of the load.

## Figures and Tables

**Table 1:** The material data for zirconia and ceramic adopted for calculation purposes.

Material	Young's Modulus	Poisson ratio
Zirconia	205 GPa	0.16
Ceramic	70 GPa	0.19

## Data Availability

The data used to support the findings of this study are available from the corresponding author upon request.
